# Impact of Zinc Supplementation on Subsequent Morbidity and Growth in Bangladeshi Children With Persistent Diarrhoea

**Published:** 2007-03

**Authors:** S.K. Roy, A.M. Tomkins, S.M. Akramuzzaman, B. Chakraborty, G. Ara, R. Biswas, K.E. Islam, W. Khatun, S.P. Jolly

**Affiliations:** ^1^ ICDDR,B, GPO Box 128, Dhaka 1000, Bangladesh; ^2^ Centre for International Child Health, Institute of Child Health, 30, Guilford Street, London WC1N 1EH, UK; ^3^ Institute of Food Science and Technology, Bangladesh Council of Scientific and Industrital Research, Dhanmondi, Dhaka 1205, Bangladesh

**Keywords:** Zinc, Zinc supplementation, Diarrhoea, Persistent, Child growth, Infant growth, Morbidity, Prospective studies, Follow-up studies, Impact studies, Bangladesh

## Abstract

This study was conducted to explore whether supplementation of zinc to children during persistent diarrhoea has any subsequent effect on morbidity and growth. A prospective follow-up study was conducted among children, aged 3–24 months, with persistent diarrhoea, who participated earlier in a double-blind randomized placebo-controlled trial. During persistent diarrhoea, children were randomly allocated to receive either zinc in multivitamin syrup or only multivitamin syrup for two weeks. After recovering from diarrhoea, 76 children in the multi-vitamin syrup and 78 children in the zinc plus multivitamin syrup group were followed up for subsequent morbidity and growth. Weekly morbidity and two-weekly anthropometric data were collected for the subsequent 12 weeks. Data showed that episodes and duration of diarrhoea were reduced by 38% and 44% respectively with supplementation of zinc. There was no significant difference in the incidence or duration of respiratory tract infection between the zinc-supplemented and the non-supplemented group. Improved linear growth was observed in underweight children (weight-for-age <70% of the National Center for Health Statistics standard) who received zinc compared to those who did not receive.

## INTRODUCTION

Diarrhoea is an important factor to precipitate malnutrition in children, and a frequent cause of growth faltering (1, 2). Associated zinc deficiency is likely to augment the process of growth faltering. Children with protein-energy malnutrition are often deficient in essential micronutrients, including zinc. Results of studies suggest that zinc deficiency is likely to be a causal factor of increased morbidity (3) and growth faltering in malnourished children (4, 5).

Zinc supplementation reduced the incidence (6), duration, and severity of diarrhoea in children (7) and had a protective effect on diarrhoea and respiratory infections (8). A study in India documented that zinc supplementation reduced the incidence of persistent diarrhoea by 73% in children with lower serum zinc concentrations (<7.65 μmol/L). Results of a study also showed a significant reduction in episodes of dysentery in male children (6). It is likely that episodes of diarrhoea result in a further zinc-deficient state in malnourished children due to the higher loss of zinc in persistent diarrhoea stool, which may lead to increased morbidity. Loss of zinc in diarrhoea stool may be as high as 159 μg/kg.day in acute diarrhoea (9) and 300 μg/kg.day in persistent diarrhoea (10).

Several studies have shown the positive effect of zinc supplementation on growth among stunted (11–13) and malnourished children. Recently, result of a meta-analysis of 25 studies showed that zinc supplementation increased height and weight in children (14). Results of another study showed that zinc supplementation for five months (10 mg/d) increased both weight and height significantly in the zinc-supplemented group compared to the non-supplemented group (11). During nutritional rehabilitation, severely-malnourished children had a greater gain in length with zinc supplementation in many countries, including Bangladesh (15). Supplementation of zinc has also led to better growth rate in preterm and full-term healthy infants compared to non-supplemented ones (4).

Although some information is available on the effect of zinc supplementation on subsequent morbidity and growth in children with acute diarrhoea (13), there is no information about any subsequent effect of zinc supplementation on morbidity and growth of children who received zinc for two weeks only during persistent diarrhoea.

We, therefore, undertook a study to follow up children who received zinc during persistent diarrhoea to explore whether there is any subsequent effect of zinc supplementation on morbidity and growth after discharge from hospital. It is hypothesized that supplementation of zinc during persistent diarrhoea will reduce subsequent morbidity and growth faltering in malnourished children.

## MATERIALS AND METHODS

### Study design

A prospective follow-up study was conducted among children aged 3–24 months, who participated earlier in a double-blind randomized placebo-controlled trial for clinical recovery from persistent diarrhoea to explore the impact of zinc supplementation on morbidity and growth after discharge from hospital.

### Participants

The participants were children of both sexes, aged 3–24 months, who were previously treated with zinc or placebo during persistent diarrhoea (16). Children who attended the Clinical Research and Service Centre (now Dhaka Hospital) of ICDDR,B, during 1987–1989, were included in the study. Children from urban and periurban areas within the 20-km radius of the Centre only were considered for the study. The exclusion criteria included the presence of any severe systemic infection, temperature more than 38 °C, and pedal oedema.

### Selection of subjects

**Fig. 1.**
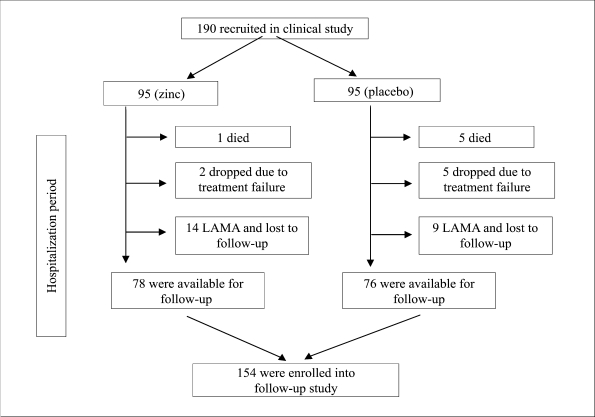
Flow-diagram of selection of subjects

Initially, 190 children with persistent diarrhoea were enrolled into the clinical study. Of these children, 154 children were available for the morbidity and growth follow-up study. The remaining 36 children (17 from the zinc group and 19 from the placebo group) were dropped from the study for various reasons (Fig. 1).

*LAMA: Left against medical advice

**Intervention:** During persistent diarrhoea, each subject received either 20 mg of elemental zinc in a multivitamin syrup daily or multivitamin alone in three-divided doses for two weeks. Syrup, manufactured by ACME Laboratories Limited, Dhaka, Bangladesh, was started immediately after collection of baseline blood and stool samples for laboratory investigations. During hospitalization, the syrup was administered by nurses and after discharge from the hospital by mothers trained during their hospital stay. Children were not discharged from the hospital until recovery, i.e. not before passing soft stool (except left without medical advice). After discharge from the hospital, trained health workers ensured daily intake of syrup through checking the remaining portion of syrup in the bottle during home-visits on alternate days.

**Anthropometry:** After discharge from the hospital, children were followed up at home once a week for the first month and then fortnightly for the next two months. During follow-up, body-weight was obtained using a Salter scale (UK) with an accuracy of 100 g. Supine-length was measured with a locally-constructed length board with a precision of 1 mm. All instruments were standardized everyday in the morning before measurements. For accuracy in weight and length, three measurements were obtained, and their average was accepted as the correct measurement. The nutritional status was determined as percent of the median National Center for Health Statistics (NCHS) standard (17). Underweight was defined as weight-for-age ≤70% of the median NCHS standard. Weekly morbidity data were collected for 12 weeks using precoded questionnaires. During weekly home-visits, mothers were asked about the exact day of the beginning of any illness, its duration, and the day of recovery. Children who were not available for follow-up on the scheduled date were visited on the following days.

**Morbidity:** Morbidity data were collected weekly during the first four weeks of follow-up and then two weekly up to 12 weeks using the recall method. A coded questionnaire was used for determining the number of episodes and the duration of each episode. The duration of episode was determined counting the days between onset of illness and recovery as described by the mother.

**Ethical consent:** The Ethical Review Committee of ICDDR,B approved the study, and a written informed consent was obtained from parents of all children.

### Definitions

**Diarrhoea:** Diarrhoea was defined as three or more liquid, watery or mucoid stools in 24 hours or any number of stools containing blood. A new episode of diarrhoea was separated from any previous episode by an interval of at least 48 hours with no diarrhoea.

**Upper respiratory tract infection:** Upper respiratory tract infection was defined by the presence of cough, nasal discharge, and respiratory rate less than 50 or 40 in infants aged less than one year and children aged 1–2 year(s) respectively.

**Lower respiratory tract infection:** Lower respiratory tract infection was defined by the presence of fast breathing (respiratory rate more than 50 per minute in infants aged less then one year, >40 per minute in infants aged 1–2 year(s), difficulty in breathing with cough, and body temperature above 38 ºC or presence of chest in-drawing.

### Laboratory investigations

**Serum zinc:** Venous blood was collected between 9 and 11 am after initial correction of dehydration on the day of selection of subjects before beginning of supplementation and again at the end of two-week's supplementation, in nitric acid-washed zinc-free glass vials for estimation of serum zinc. Serum zinc was measured using an atomic absorption spectrophotometer (AAS, Perkin-Elmer Model 3100) according to Prasad *et al.* (18).

### Statistical analysis

Subjects were taken for growth and morbidity analysis from the beginning of follow-up. The effect of zinc supplementation was compared between the groups. As the effect of zinc is more pronounced in malnourished children, subgroup analysis was restricted to underweight children (≤70% weigh-for-age). Student's *t*-test was used for comparing the means of net gain in length between the two groups. Gain in weight and length was compared between the groups using the *t*-test. The episodes and duration of diarrhoea and respiratory infections were compared between the groups using *t*-tests. Data were analyzed using the SPSS/PC+ and NCHS statistical packages. Statistical significance was accepted at 5% probability level.

## RESULTS

In total, 154 children were followed up for 12 weeks after two weeks of zinc supplementation during persistent diarrhoea. Of the 154 children, 76 received multivitamin syrup only, and 78 received zinc with multivitamin syrup during persistent diarrhoea before enrollment into the follow-up study. Seven (5%) children did not complete the 12-week follow-up study period due to migration. Four subjects migrated from the placebo group, and three subjects migrated from the zinc group. Those who migrated had comparable baseline characteristics, such as anthropometric measurements and initial duration of diarrhoea with the remaining children of their respective trial group. Children in the placebo and zinc-supplemented groups were comparable on admission for age, sex, duration of diarrhoea, nutritional status, and levels of serum zinc (Table 1). The subjects were also comparable by their feeding status; an equal proportion of children was breastfed in the control or zinc group (49% vs 51%, p=0.78, data not shown). Table 2 shows baseline comparison in characteristics of the under-weight subgroup.

**Table 1.** Comparison of selected baseline characteristics of study subjects (mean±SD)

Characteristics	Zinc (n=78)	Placebo (n=76)	p value
Age (months)	7.0±3.0	8.0±4.0	0.31
Sex (M/F)	56/22	45/31	0.10
Weight-for-age (z-score)	-2.46±1.0	-2.44±1.2	0.18
Height-for-age (z-score)	-1.64±1.3	-1.44±1.1	0.29
Weight-for-height (z-score)	-1.81±1.0	-1.68±0.86	0.06
MUAC (cm)	11.7±1.0	11.6±1.0	0.71
Duration (days) of diarrhoea on admission	23±10	22±12	0.73
Serum zinc (μmol/L)	13.4±4.8	13.2±4.1	0.10

MUAC=Mid-upper arm circumference;

F=Female;

M=Male;

SD=Standard deviation

**Table 2.** Baseline characteristics of underweight children (weight-for-age <70% of NCHS median)

Characteristics	Zinc (n=36) mean±SD	Placebo (n=32) mean±SD	p value
Age (months)	7.6±3.1	9.3±3.7	0.04
Weight-for-age (z-score)	-3.3±0.7	-3.6±0.8	0.08
Height-for-age (z-score)	-2.6±0.8	-2.3±0.1	0.18
Serum zinc (μmol/L)	13.9±4.5	13.4±3.8	0.62
Diarrhoea on admission (duration in days)	25.0±10.9	22.0±9.9	0.24

NCHS=National Center for Health Statistics;

SD=Standard deviation

During the three-month follow-up period, 26 of the 78 children (33%) in the zinc group experienced at least one episode of diarrhoea compared to 33 of the placebo group (33/76; 43%). The mean diarrhoeal episodes were significantly less in the zinc-supplemented children compared to the control children (0.53±0.10, vs 0.86±0.14, p<0.05) (Table 3). The mean duration of subsequent diarrhoea episodes in the zinc group was 44% shorter compared to that of the control group (1.9±0.40 vs 3.4±0.62 days, p<0.05) (Table 3). Zinc supplementation was not associated with the incidence or duration of respiratory infections during the follow-up period.

**Table 3.** Comparison of illness episodes and duration between groups in patients with persistent diarrhoea during the follow-up (mean±SEM)

Illness	Zinc (n=36) mean±SD	Placebo (n=32) mean±SD	p value
Diarrhoea (no. of episodes)	0.53±0.10	0.86±0.14	0.05
Duration (days) of diarrhoea	1.9±0.40	3.4±0.62	0.05
RTI (no. of episodes)	1.42±0.14	1.54±0.86	0.55
Duration (days) of RTI Student's *t*-test	12.4±1.64	12.0±1.72	0.92

RTI=Respiratory tract infection;

SD=Standard deviation;

SEM=Standard error of the mean

**Fig. 2.**
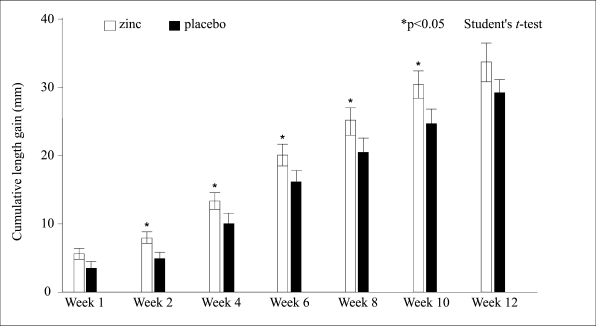
Impact of zinc supplementation on linear growth among lighter children (weight-for-age ≤70%) (mean±SEM)

During the subsequent 12 weeks of follow-up, cumulative gain in length was comparable between the zinc-supplemented and the control children when data from all patients were compared (35.7±12.9 mm vs 31.2±12.6, p=0.6; data not shown). Hence, when the gain in length was compared among underweight children (weight-for-age, ≤70% of NCHS, n=68), a greater cumulative gain in length (24%) was observed in the zinc-supplemented group (30.2±10.0 vs 24.4±10.8, p<0.03) compared to the control group. The accelerated length velocity of underweight subjects continued from the second week to 10th week of follow-up (p=0.03) (Fig. 2). There was no significant difference in cumulative gain in body-weight between the zinc and the control group as a whole or in the subgroups.

SEM=Standard error of the mean

**Mortality:** In total, 13 children died during the 12- weeks period. Of these children, six died in the zinc-supplemented group, and seven died in the placebo group. Of these deaths, two died in week 1, four died in week 2, three died in week 3, one died in week 5, and three died in week 6. No deaths occurred between week 6 and 12. Causes of death diagnosed by verbal autopsy included bronchopneumonia, septicaemia, and severe diarrhoea. Two patients with persistent diarrhoea returned to the hospital for treatment, and both of them had both diarrhoea and pneumonia, and one died of severe intractable diarrhoea.

## DISCUSSION

The study examined the effect of supplementation of zinc in patients with persistent diarrhoea on the reduction of subsequent morbidity due to diarrhoea and respiratory tract infections and growth faltering.

The clinical benefits of zinc supplementation in these children with persistent diarrhoea were early recovery especially in underweight and male children described earlier (16). A significantly higher number of children also dropped out from the placebo group due to prolonged suffering. Results of our earlier studies in acute diarrhoea showed the benefits of zinc supplementation to malnourished children, such as reduced stool output and reduced the duration of recovery (19). Home follow-up of those acute diarrhoea children after discharge showed subsequent benefit of increased linear growth among those who received zinc during diarrhoea (13). The follow-up also showed significantly reduced respiratory tract infection in stunted children who received zinc during acute diarrhoea.

The results of the present study showed that there were carry-over benefits of zinc supplementation in addition to early recovery of persistent diarrhoea, which extended for about three months after supplementation.

The zinc-supplemented patients with persistent diarrhoea had fewer attacks of diarrhoea, possibly due to their better immune status after supplementation of zinc. Diarrhoea illness may be prolonged by factors that cause injury to intestinal mucosa or delay in repair of mucosa. Our earlier study demonstrated that zinc has profound effects on intestinal epithelial tissue in respect of growth of mucosal tissue and ultrastructures which are directly related to water and electrolyte transport (20).

In the present study, reduction in diarrhoea attacks (38%) and reduction in the duration of episodes (44%) in the zinc-supplemented children have a similarity with a community-based study conducted in India where children with acute diarrhoea had a 7% reduction in probability of continuing diarrhoea beyond seven days and 30% reduction in subsequent diarrhoea episodes with zinc supplementation (7). Our results have further confirmed results of a zinc-supplementation study on growth-retarded Vietnamese children who had a three-fold decrease in the incident of diarrhoea and a 2.5-fold decrease in respiratory infections during a five-month period of zinc supplementation (11). The supplementation of zinc in that study, however, was continuous and for a much longer period compared to our study. The effect of reduction in diarrhoea episodes was about 1.5-fold in our study, and the decrease in duration of episodes was also similar. The results of the present study are consistent with the results of studies conducted in free-living children having a reduction in diarrhoeal illness following zinc supplementation in Mexico (21) and Guatemala (22). In the Mexican study, both diarrhoea and respiratory infection decreased significantly with a 20-mg daily zinc supplementation for 12 months compared to placebo, but the effect was less pronounced in respiratory infections compared to diarrhoea (23).

The effect of zinc on linear growth was not evident when all children (both malnourished and well-nourished) of both the groups were compared; it was, however, evident in more malnourished children. Several points are to be considered in this respect, such as zinc increases growth in a zinc-deficient state acting as a rate-limiting factor, and a larger proportion (60%) of our subjects were malnourished. The carry-over growth effect of zinc during short supplementation in persistent diarrhoea was reflected in the more-deficient group, i.e. underweight subjects, but not in the well-nourished children.

The zinc-supplemented children had reduced episodes of diarrhoea and, at the same time, had additional gain in length. Although diarrhoea is a potential confounder on the effect of zinc on growth in this study, the effect of zinc supplementation on gain in length was independent of the effect of zinc on diarrhoea. Supplemental zinc had been exhausted during growth as a rate-limiting factor and did not support increased growth after 10 weeks. Growth-retarded Vietnamese children had increased growth with zinc supplementation for three months with increased insulin-like growth factor I (11). This result suggests that zinc has a particular role in stimulating growth by activating growth hormone. The mechanism of zinc to facilitate skeletal growth is not fully understood, but alkaline phosphatase is a zinc metalloenzyme, which aids in bone growth. Zinc also participates in bone growth via enhanced mineralization (24).

Data on gain in length during supplementation of zinc are dependent on the level of malnutrition in the children and the dietary quality. Results of studies of zinc supplementation during rehabilitation of severely-malnourished children showed gain in length when children were fed high-energy and high-protein diets (15, 25, 26). In our study, children went home with advice to take the usual home-diet during the follow-up period, and they were not given zinc during the follow-up period. Plausible causes of failure to increase gain in body-weight in our study would include that the children were suffering from persistent diarrhoea with significant malabsorption of nutrients (27). Zinc is excreted in diarrhoeal stool leading to a risk of a negative zinc balance (9).

No difference in respiratory infections was, however, observed between the two groups. The supplementation of zinc was not continued during the follow-up period to see the effect of supplementation as in other studies. It was rather explored whether there was any carry-over effect of zinc during the subsequent period after persistent diarrhoea. Even if there were any carry-over effect of zinc on respiratory infection, our sample size was not adequate to detect the significant difference between the zinc-supplemented and the non-supplemented groups.

The results of our study indicate that supplementation of zinc to children during persistent diarrhoea has the additional benefit of reducing subsequent diarrhoea episodes and their duration in all children for about three months and overcoming growth faltering among underweight children. These results have a particular significance for public-health policy. We, therefore, recommend supplementation of zinc at a dose of 20 mg/day for at least two weeks in all children during persistent diarrhoea. Zinc mixed with oral rehydration solution (ORS) could be another possibility for supplementation of zinc during diarrhoea, but the appropriate dose of zinc per litre of ORS needs to be determined through a well-designed controlled clinical trial.

## ACKNOWLEDGEMENTS

This study was supported by the Wellcome Trust (UK) and ICDDR,B. ICDDR,B is supported by countries and agencies which share its concern for the health problems of developing countries. The authors gratefully acknowledge the statistical advice of Dr. Abbas Bhuiya. The authors also acknowledge Ms Jarin Sultana, Ms Azmira Begum, and Ms Sabira Islam for their dedicated services during the study and Mr. M.A. Wahed for his assistance with biochemical analysis.
